# Radical Resection of a Late-Relapsed Testicular Germ Cell Tumour: Hepatectomy, Cavotomy, and Thrombectomy

**DOI:** 10.1155/2014/713049

**Published:** 2014-12-21

**Authors:** C. Ní Leidhin, C. E. Redmond, A. M. Cahalane, H. M. Heneghan, R. Motyer, E. R. Ryan, E. Hoti

**Affiliations:** Departments of Hepatobiliary Surgery and Radiology, The National Liver Transplant Unit, St. Vincent's University Hospital, Elm Park, Dublin 4, Ireland

## Abstract

Up to 3.2% of patients with testicular germ cell tumours represent with late-relapsing disease. Aggressive surgical resection confers the greatest chance of cure in this patient group. We present the case of a late and extensively relapsed nonseminomatous germ cell tumour with thrombus present along the entire length of the inferior vena cava, as well as in the right hepatic vein. Techniques practised in liver transplantation were used to achieve complete resection of the tumour thrombus. This case illustrates the enhanced potential for tumour resection through a fusion of principles derived from surgical oncology and liver transplantation.

## 1. Introduction

Testicular cancer is the most common solid organ malignancy in males between the ages of 15 and 35 [1] and its incidence is increasing steadily [2]. Approximately 95% of malignant testicular cancers are germ cell tumours (GCTs) [3]. Of these, more than 50% are mixed [4]. GCTs that contain both seminomatous and nonseminomatous elements are managed as nonseminomatous germ cell tumours (NSGCTs), since these behave more aggressively clinically [3]. Late relapse of a malignant GCT, defined as disease recurrence more than two years after successful treatment, occurs in up to 3.2% of patients [5]. The most common site for relapse is the retroperitoneal space [5]. Aggressive surgical resection is advocated in cases of late relapse, conferring the greatest chance of patient cure [6]. We report the case of a late-relapsed NSGCT with an unusually extensive distribution of disease recurrence, referred to the National Liver Transplant Unit of Ireland for potential surgical resection.

## 2. Case Report

A 42-year-old man presented with a right testicular mass and elevated *α*-fetoprotein and lactate dehydrogenase levels of 300 ng/mL and 1781 IU/L, respectively. Staging computed tomography (CT) scan demonstrated retroperitoneal lymphadenopathy. Orchidectomy was performed with histopathological analysis of resected tissue revealing a mixed seminomatous and nonseminomatous GCT, pT2N1M0S2, stage IIIb disease [7, 8], International Germ Cell Consensus Classification intermediate-prognosis group [9]. The patient underwent systemic chemotherapy with four cycles of etoposide and carboplatin, followed by retroperitoneal lymph node dissection.

**Figure 1:**
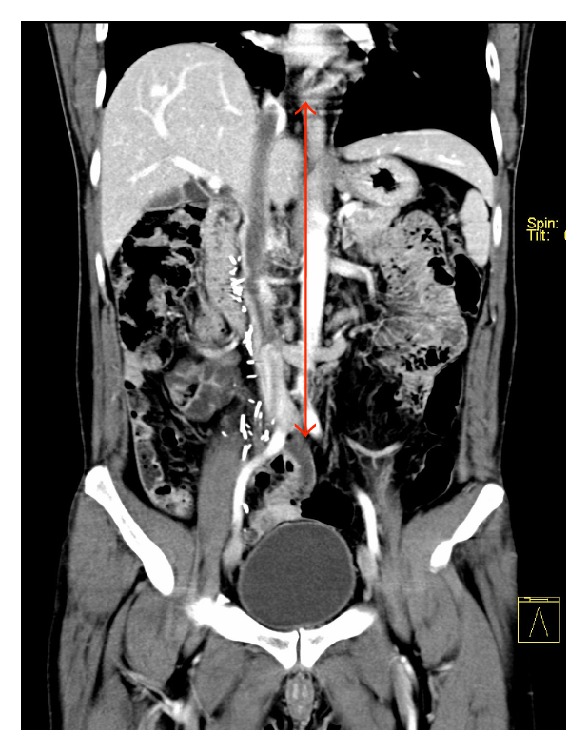
Reformatted coronal CT image demonstrating contiguous tumour thrombus in the inferior vena cava extending to the junction of the right atrium.

**Figure 2:**
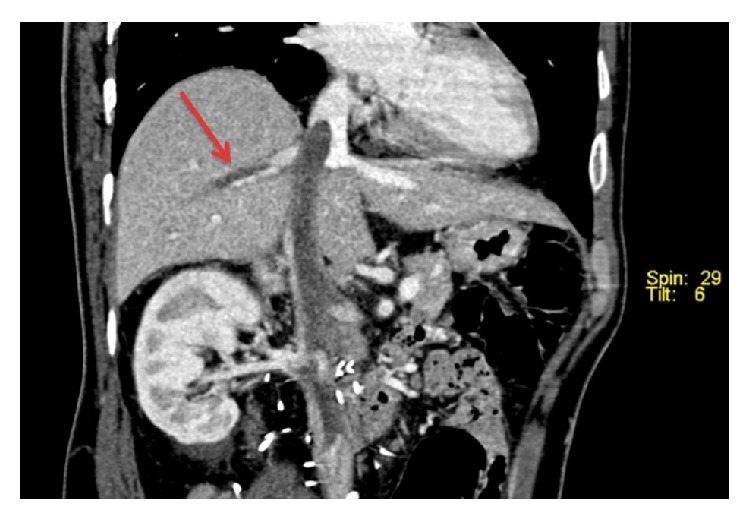
Reformatted coronal CT image demonstrating tumour thrombus in the inferior vena cava, extending into the right hepatic vein.

He was well until six years after treatment, when he represented with general malaise and bipedal oedema. A CT scan demonstrated contiguous thrombus in the inferior vena cava (IVC) extending to the junction of the right atrium (RA) (Figure 1) and involving the right hepatic vein (RHV) (Figure 2) as well as enlarged aortocaval lymph nodes, suspicious for metastatic disease. Biopsy of the thrombus confirmed recurrent combined GCT.

The patient was referred to our department for assessment regarding potential for thrombectomy. Following multidisciplinary case review and patient counselling, it was decided to attempt surgical resection.

**Figure 3:**
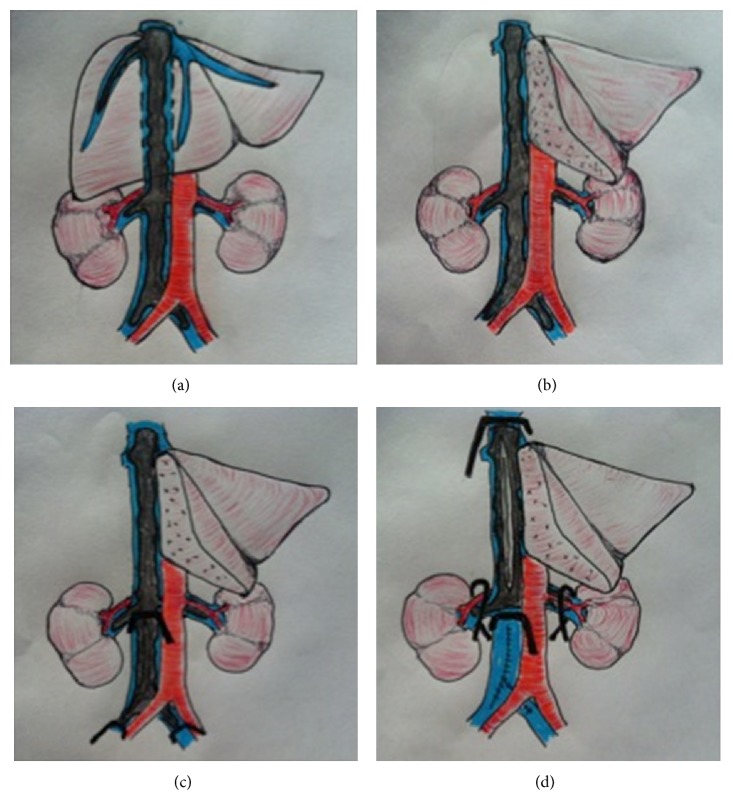
Schematic illustration demonstrating (a) IVC exposure and the extent of the tumour thrombus prior to resection. (b) Retrohepatic VC exposure via a right hepatectomy. (c) Infrarenal VC occlusion, enabling cavotomy and thrombus extraction. (d) Suprarenal VC occlusion, enabling cavotomy and thrombus extraction.

At laparotomy, the entire length of the IVC was exposed (Figure 3(a)). First, exposure of the retrohepatic vena cava was achieved via a right hepatectomy (Figure 3(b)). This was necessary for complete excision of the RHV tumour thrombus. The liver hanging manoeuvre [10] was used in order to minimise manipulation of the IVC/RHV and to prevent tumour fragmentation and/or embolisation. The ascending colon and duodenum were mobilised to expose the infrahepatic VC. The degree of extension of the tumour thrombus necessitated that the suprahepatic VC be controlled at the level of the RA. Complete dissection of the diaphragm around the VC was required. The renal veins and arteries were then controlled, followed by the VC above the iliac bifurcation. Control of and access to the inferior mesenteric vein (IMV) was obtained for venovenous bypass (VVBP). Thrombectomy was commenced at the infrarenal portion of the VC. Occlusion of the infrarenal VC was achieved and cavotomy and thrombus extraction was performed (Figure 3(c)). The infrarenal clamp was then briefly repositioned above the renal veins and the tumour thrombus was extracted from the renal veins (Figure 3(d)). Occlusion of the renal arteries was not required. The clamp was then repositioned below the renal veins and the cavotomy was closed. Total vascular exclusion (TVE) of the liver remnant was used to enable cavotomy and thrombectomy of the retrohepatic and suprahepatic VC with maximal haemorrhage control. Portal flow was diverted via a cannula placed in the IMV. Systemic blood flow was diverted through a cannula placed in the right femoral vein via Seldinger technique. Blood returned to the systemic circulation via a cannula sited in the left internal jugular vein. In addition to ensuring adequate cardiac return, VVBP prevented congestion of the portal system and avoided clamping of the renal vessels, thereby minimising the potential for ischaemia. Once the thrombus was extracted completely, the VC was closed. Finally, dissection of enlarged paracaval and aortocaval lymph nodes was performed.

The surgery lasted six and a half hours in total. VVB was used for a total of 45 minutes. Estimated intraoperative blood loss was 14,000 mLs. The patient received 17 units of packed red blood cells (pRBC), two pools of platelets, four units of Octaplas, and six grams of fibrinogen during the procedure. He was transferred to the intensive care unit postoperatively, where he spent the next three days.

His recovery was uneventful. No hepatic or renal impairment was observed in the postoperative period. There was no chyle leak. The patient was transfused one further pool of platelets and two units of pRBCs prior to his discharge home day 17 postoperatively.

Histopathological analysis of resected thrombus and one infrarenal lymph node revealed metastatic NSGCT.

The patient is monitored in accordance with the European Society of Medical Oncology guidelines [11] and remained disease- and symptom-free two years postoperatively.

## 3. Discussion

Renal malignancies account for up to 70% of IVC tumour thrombi [12] but this phenomenon is also seen with other genitourinary tumours, that is, GCTs. Intracaval tumour thrombus development per continuitatem from a primary testicular tumour has been described in several case series [13–15]. To the best of our knowledge, this is the first reported case of IVC/hepatic vein involvement by a relapsed NSGCT.

Venous congestion secondary to IVC tumour thrombus can cause significant lower extremity oedema, varicocele, pulmonary emboli, and/or Budd-Chiari syndrome [16].

Surgical thrombectomy is the most effective treatment option. If successful, it relieves symptoms and improves long-term survival [17].

This case presented a complex surgical challenge. The major risks identified during the preoperative planning were those of major haemorrhage and tumour fragmentation [18, 19]. Techniques practiced in liver transplantation were adapted to access and achieve vascular control of the retrohepatic IVC, thereby enabling satisfactory tumour resection while maintaining the patient's haemodynamic stability. Our operative approach ensured minimal tumour manipulation, with proximal and distal IVC control throughout the operation preventing embolisation of tumour fragments. In this case, extension of the tumour thrombus into the RHV posed yet another technical challenge, necessitating innovative surgery, including a right hepatectomy.

This case emphasises the important and ever-evolving role of radical surgery for symptom palliation and improved survival in patients with late-relapsing GCTs. It illustrates how a fusion of principles derived from surgical oncology and liver transplantation may enhance the potential for tumour resection.

This type of radical and technically challenging surgery should only be carried out, following multidisciplinary case review, by experienced surgeons in high-volume, highly specialised tertiary centres, something that has been shown to significantly reduce perioperative mortality and local recurrence rates [20, 21].
